# Author Correction: MusMorph, a database of standardized mouse morphology data for morphometric meta-analyses

**DOI:** 10.1038/s41597-023-02320-x

**Published:** 2023-06-28

**Authors:** Jay Devine, Marta Vidal-García, Wei Liu, Amanda Neves, Lucas D. Lo Vercio, Rebecca M. Green, Heather A. Richbourg, Marta Marchini, Colton M. Unger, Audrey C. Nickle, Bethany Radford, Nathan M. Young, Paula N. Gonzalez, Robert E. Schuler, Alejandro Bugacov, Campbell Rolian, Christopher J. Percival, Trevor Williams, Lee Niswander, Anne L. Calof, Arthur D. Lander, Axel Visel, Frank R. Jirik, James M. Cheverud, Ophir D. Klein, Ramon Y. Birnbaum, Amy E. Merrill, Rebecca R. Ackermann, Daniel Graf, Myriam Hemberger, Wendy Dean, Nils D. Forkert, Stephen A. Murray, Henrik Westerberg, Ralph S. Marcucio, Benedikt Hallgrímsson

**Affiliations:** 1 Alberta Children’s Hospital Research Institute, University of Calgary, 28 Oki Dr NW, Calgary, AB T3B 6A8 Canada; 2 The McCaig Institute for Bone and Joint Health, University of Calgary, 3280 Hospital Dr NW, Calgary, AB T2N 4Z6 Canada; 3 Department of Cell Biology and Anatomy, Cumming School of Medicine, University of Calgary, 3330 Hospital Dr NW, Calgary, AB T2N 4N1 Canada; 4 Department of Biology, McMaster University, 1280 Main St W, Hamilton, ON L8S 4L8 Canada; 5 School of Dental Medicine, University of Pittsburgh, 3501 Terrace St, Pittsburgh, PA 15213 USA; 6 Orthopaedic Trauma Institute, ZSFG, UCSF, 2550 23rd St, San Francisco, CA 94110 USA; 7 Department of Biological Sciences, University of Calgary, 2500 University Dr NW, Calgary, AB T2N 1N4 Canada; 8 Center for Craniofacial Molecular Biology, Department of Biomedical Sciences, Herman Ostrow School of Dentistry, University of Southern California, Los Angeles, 2250 Alcazar St, Los Angeles, CA 90033 USA; 9 Department of Biochemistry and Molecular Medicine, Keck School of Medicine, University of Southern California, Los Angeles, 1975 Zonal Ave, Los Angeles, CA 90033 USA; 10 Department of Biochemistry and Molecular Biology, Cumming School of Medicine, University of Calgary, 3330 Hospital Dr NW, Calgary, AB T2N 4N1 Canada; 11 Institute for Studies in Neuroscience and Complex Systems (ENyS) CONICET, Av. Calchaquí, 5402 Florencio Varela, Buenos Aires Argentina; 12 Information Sciences Institute, Viterbi School of Engineering, University of Southern California, 4676 Admiralty Way, Marina del Rey, CA 90292 USA; 13 Department of Comparative Biology and Experimental Medicine, Faculty of Veterinary Medicine, University of Calgary, 3330 Hospital Dr NW, Calgary, AB T2N 4N1 Canada; 14 Department of Anthropology, Stony Brook University, 100 Nicolls Rd, Stony Brook, NY 11794 USA; 15 Department of Craniofacial Biology, University of Colorado Anschutz Medical Campus, 12801 East 17th Ave, Aurora, CO 80045 USA; 16 Department of Molecular, Cellular and Developmental Biology, University of Colorado Boulder, Boulder, CO 80309 USA; 17 Department of Anatomy and Neurobiology, University of California, Irvine, Irvine, CA 92697 USA; 18 Center for Complex Biological Systems, University of California, Irvine, Irvine, CA 92697 USA; 19 Department of Developmental and Cell Biology, University of California, Irvine, Irvine, CA 92697 USA; 20 Environmental Genomics and Systems Biology Division, Lawrence Berkeley National Laboratory, 1 Cyclotron Rd, Berkeley, CA 94720 USA; 21 U.S. Department of Energy Joint Genome Institute, Lawrence Berkeley National Laboratory, 1 Cyclotron Rd, Berkeley, CA 94720 USA; 22 School of Natural Sciences, University of California, Merced, 5200 Lake Rd, Merced, CA 95343 USA; 23 Department of Biology, Loyola University Chicago, 1032 W Sheridan Rd, Chicago, IL 60660 USA; 24 Department of Orofacial Sciences and Program in Craniofacial Biology, University of California, San Francisco, 513 Parnassus Ave, San Francisco, CA 94143 USA; 25 Department of Pediatrics and Institute for Human Genetics, University of California, San Francisco, 513 Parnassus Ave, San Francisco, CA 94143 USA; 26 Department of Pediatrics, Cedars-Sinai Medical Center, 8700 Beverly Blvd, Los Angeles, CA 90048 USA; 27 Department of Life Sciences, Faculty of Natural Sciences, The Ben-Gurion University of the Negev, David Ben Gurion Blvd 1, Be’er Sheva, Israel; 28 Department of Archaeology, University of Cape Town, Rondebosch, Cape Town, 7700 South Africa; 29 Human Evolution Research Institute, University of Cape Town, Rondebosch, Cape Town, 7700 South Africa; 30 School of Dentistry, Faculty of Medicine and Dentistry, University of Alberta, 116 St. and 85 Ave, Edmonton, AB T6G 2R3 Canada; 31 Department of Medical Genetics, Faculty of Medicine and Dentistry, University of Alberta, 116 St. and 85 Ave, Edmonton, AB T6G 2R3 Canada; 32 Department of Radiology, Cumming School of Medicine, University of Calgary, 3330 Hospital Dr NW, Calgary, AB T2N 4N1 Canada; 33 The Jackson Laboratory, 600 Main St, Bar Harbor, ME 04609 USA; 34 Department of Bioimaging Informatics, MRC Harwell Institute, Oxfordshire, OX11 0RD UK

**Keywords:** Development, X-ray tomography, Bone

Correction to: *Scientific Data* 10.1038/s41597-022-01338-x, published online 25 May 2022

The order of the column headings was incorrect in Table 1, meaning values were listed under the wrong descriptions. This has been corrected in the pdf and HTML versions of the article.

