# Supplementary material for: MusMorph, a database of standardized mouse morphology data for morphometric meta-analyses
Source: Sci Data. 2022 May 25;9:230. doi: 10.1038/s41597-022-01338-x (PMC9133120; doi:10.1038/s41597-022-01338-x)
Supplement: Supplementary file 1 — Supplementary Data [file 41597_2022_1338_MOESM1_ESM.pdf]

Table of Contents

Supplementary Table 1 2
Supplementary Table 2 4
Supplementary Table 3 5
Supplementary Table 4 6
Supplementary Table 5 8
Supplementary Table 6 9
Supplementary Figure 1 10
Supplementary Figure 2 11
Supplementary Figure 3 12
Supplementary Figure 4 13

Table S1. Summary of MusMorph datasets on FaceBase. Dataset is the name of the dataset. Stage(s) are the development stages within the dataset. N is the sample size. Strain(s) and Genotype(s) are the number of unique strains and genotypes. +/+, +/-, -/-, Other, and Unknown are the number of wildtype, heterozygous, homozygous, other (e.g., neo/null), and unknown specimen zygosities, respectively. F, M, and Unknown are the number of females, males, and unknown sexes. FaceBase DOI is the individual dataset DOI.

					Zygosity					Sex			
**Dataset**	**Stage(s)**	**N**	**Strain(s)**	**Genotype(s)**	**+/+**	**+/-**	**-/-**	**Other**	**Unknown**	**F**	**M**	**Unknown**	**FaceBase DOI**
Ap2	E10.5, E11.5	125	1	5	54	×	×	68	3	×	×	125	doi.org/10.25550/3-JQMG
B9d	E11.5, Adult	132	1	4	17	63	31	×	21	19	×	113	doi.org/10.25550/3-JQMM
Nosip	E10.5, Adult	40	1	4	20	13	6	×	1	19	10	11	doi.org/10.25550/3-JQMP
Mks	E10.5, E11.5	15	1	3	6	6	3	×	×	×	×	15	doi.org/10.25550/3-JVDW
Ift	E10.5, Adult	147	2	4	38	103	1	×	5	52	×	95	doi.org/10.25550/3-JVE0
Tctn	E10.5, E11.5	51	2	8	8	24	11	×	8	×	×	51	doi.org/10.25550/3-JVE2
Shh	E11.5, Adult	219	1	5	92	125	×	×	2	36	65	118	doi.org/10.25550/3-JVE6
Fgf	E10.5, E11.5, Adult	250	1	8	95	90	23	×	42	24	33	193	doi.org/10.25550/3-JVEM
Bulgy	E10.5, E11.5, Adult	262	1	4	99	139	1	×	23	105	71	86	doi.org/10.25550/3-JZ9G
Strain Comparison	E11.5, Adult	244	10	9	219	×	×	×	25	103	107	34	doi.org/10.25550/3-JZ9J
Wnt	E10.5, E11.5	136	4	1	×	×	×	×	136	×	×	136	doi.org/10.25550/3-JZ9Y
Placenta	E14.5	84	1	8	33	20	31	×	×	38	24	22	Unreleased
IMPC	E15.5, E18.5	3001	1	419	881	36	2084	×	×	1387	1418	196	doi.org/10.25550/3-JZA6

Spry	Adult	260	3	9	88	114	58	×	×	108	152	×	doi.org/10.25550/3-JZAM
Osteo Imperfecta	Adult	29	1	2	29	×	×	×	×	16	13	×	doi.org/10.25550/3-KB00
Enhancer	Adult	465	3	17	138	55	270	×	×	208	254	3	doi.org/10.25550/3-KB02
Ghrhr	Adult	394	1	2	×	105	289	×	×	57	87	250	doi.org/10.25550/3-KB08
Nipbl	Adult	59	1	2	37	22	×	×	×	10	49	×	doi.org/10.25550/3-KB0J
Collaborative Cross	Adult	1129	60	58	1129	×	×	×	×	538	591	×	doi.org/10.25550/3-KB0W
BBDS	Adult	24	1	5	6	11	7	×	×	17	7	×	Unreleased
Brachymorph	Adult	30	1	1	×	×	30	×	×	14	10	6	doi.org/10.25550/3-KB1W
Hybrid	Adult	817	20	19	817	×	×	×	×	444	373	×	doi.org/10.25550/3-KB32
Brain-Face	Adult	141	2	6	×	40	74	26	1	75	66	×	doi.org/10.25550/3-KB3J
RASopathy	Adult	39	1	2	21	×	18	×	×	20	19	×	Unreleased
Bmp	Adult	274	1	24	115	131	26	2	×	121	150	3	doi.org/10.25550/3-KB46
Lrp	Adult	9	1	1	×	9	×	×	×	9	×	×	doi.org/10.25550/3-KB4J
Diversity Outbred	Adult	1048	1	8	1048	×	×	×	×	574	337	137	doi.org/10.25550/3-KB4P
Longshanks	Adult	446	1	5	446	×	×	×	×	246	200	×	doi.org/10.25550/3-KFBE
MPS	Adult	45	1	1	×	×	45	×	×	12	28	5	doi.org/10.25550/3-KFBY
Nabo	Adult	90	1	2	×	43	47	×	×	39	51	×	Unreleased
Pten	Adult	26	1	2	2	×	24	×	×	12	14	×	doi.org/10.25550/3-KFZJ
Trp	Adult	25	1	1	×	25	×	×	×	23	2	×	doi.org/10.25550/3-KFZW

Table S2. Sparse embryo anatomical landmarks/derivatives and their definitions (* indicates landmarks that are specific to the E14.5, E15.5, and E18.5 embryos).

**Paired Landmarks (R/L)**	**Anatomical Definition**
1/2	Caudal most junction of the lateral nasal process and maxillary process
3/4	Nasal aperture and rostral ventral most junction of the lateral nasal process and maxillary process
5/6	Corner of the mouth
7/8	Medial, rostral, dorsal corner of the mandibular process
11/13	Dorso-caudal most point of the lateral nasal process
12/14	Center of the eye
15/16	Dorsal most point of the nasal aperture
21/22	Junction between the bulge of the trigeminal ganglion and pontine flexure of the developing brain
23/24*	Dorso-caudal corner of whisker row
**Midline Landmarks**	**Anatomical Definition**
9	Rostral midline point of the mandibular processes
10	Rostral most point at the midline of the medial nasal processes.
17	Midline dorsal most extent of the face
18	Dorsal midline junction between the growing forebrain and midbrain lobes
19	Dorso-caudal most point on the midline of the midbrain
20	Caudal most midline point at the back of the head, just ventral to the midbrain
25*	Tip of the nose and rostral most point of the face

Table S3. Embryo landmark patch vertices, their size, the number of semilandmarks, and their row position in an individual array. Due to the extra sparse landmarks for E14.5-15.5 and E18.5, their patch positions are given in parentheses "()".

Vertex 1	Vertex 2	Vertex 3	Patch Size	No. of Semis	Rows in Array
20	19	21	10	36	23:58 (26:61)
20	19	22	10	36	59:94 (62:97)
18	19	21	10	36	95:130 (98:133)
18	19	22	10	36	131:166 (134:169)
18	14	22	10	36	167:202 (170:205)
18	12	21	10	36	203:238 (206:241)
5	12	21	10	36	239:274 (242:277)
17	12	18	10	36	275:310 (278:313)
17	14	18	10	36	311:346 (314:349)
6	14	22	10	36	347:382 (350:385)
6	14	16	7	15	383:397 (386:400)
5	12	15	7	15	398:412 (401:415)
5	7	9	5	6	413:418 (416:421)
6	8	9	5	6	419:424 (422:427)
13	16	17	5	6	425:430 (428:433)
11	15	17	5	6	431:436 (434:439)
15	16	17	4	3	437:439 (440:442)
3	10	15	4	3	440:442 (443:445)
4	10	16	4	3	443:445 (446:448)
7	8	9	4	3	446:448 (449:451)
10	15	16	4	3	449:451 (452:454)

Table S4. Sparse adult craniofacial landmarks and their definitions.

Paired Landmarks (R/L)	Anatomical Definition
2/1	Superior point of post-tympanic hook
4/3	Paroccipital process
6/5	Posterior point on internal pterygoid process
14/13	Lateral point on frontal suture
16/15	Lateral zygomatic-frontal suture
18/17	Posterior zygomaticofrontal junction
20/19	Posterior margin of malar process
22/21	Frontal-temporal-parietal junction
23/24	Anterior margin of incisive foramen
25/26	Medial maxilla-premaxilla junction
27/28	Anterior inferior zygomatic
29/30	Anterior temporo-zygomatic junction
31/32	Anterior superior alveoli
33/34	Posterior incisive foramen
35/36	Point along palatine-maxillary suture
37/38	Medial palatal-pterygoid junction
39/40	Posterior superior alveoli
41/42	Lateral palatal-pterygoid junction
43/44	Spheno-occipital synchondrosis
45/46	Anterior foramen ovale
47/48	Posterior temporo-zygomatic junction
49/50	Auditory-temporal-sphenoid junction
51/52	Anterior inferior auditory bulla
53/54	Occipital-auditory-sphenoid junction
55/56	Point along occipitomastoid suture
57/58	Medial occipital condyle
59/60	Anterior nasal and premaxilla
61/68	Frontal suture on orbital rim
62/69	Superior temporo-zygomatic suture
63/70	Posterior zygomatic process
64/71	Superior posterior tympanic ring
65/72	Occipital-auditory junction
66/67	Midline superior incisor
91/79	Posterior tympanic ring
80/81	Anterior inferior maxilla
82/85	Medial point of first upper molar
83/86	Medial point of second upper molar
84/87	Medial point of third upper molar

89/90	Superior lateral point of paroccipital process
92/93	Superior lateral most point of occipital condyle
**Midline Landmarks**	**Anatomical Definition**
7	Posterior point of presphenoid
8	Superior most point of foramen magnum
9	Posterior most point of occipital
10	Lambda
11	Bregma
12	Nasion
73	Anterior foramen magnum
74	Midline junction between the basioccipital and sphenoid
75	Midline junction between the sphenoid and presphenoid
76	Anterior junction of the endocranial presphenoid
77	Endocranial junction between the frontal and ethmoid
78	Anterior most point of nasal bone
88	Anterior point on alveolar process between incisors

Table S5. Sparse adult endocast landmarks and their definitions.

**Paired Landmarks (R/L)**	**Anatomical Definition**
5/4	Trigeminal nerve
7/6	Lateral junction between cerebellum and medulla
9/8	Lateral junction between occipital lobe and cerebellum
**Midline Landmarks**	**Anatomical Definition**
1	Distal most point of olfactory bulb
2	Midline junction between olfactory bulb and anterior olfactory nucleus
3	Midline junction between anterior olfactory nucleus and ventral striatum, optic nerves
10	Midline junction between midbrain and cerebral cortex
11	Midline junction between olfactory bulb and cerebral cortex
12	Optic chiasma

Table S6. Sparse adult mandible landmarks and their definitions.

Paired Landmarks (R/L)	Anatomical Definition
1/6	Posterior mandibular angle
2/7	Posterior mandibular condyle
3/8	Posterior superior most point of coronoid process
4/9	Mandibular tuberosity and posterior point of molar alveolar rim
5/10	Anterior point of molar alveolar rim
13/14	Inferior most point of mental protuberance
15/18	Mandibular foramen/inferior alveolar foramen
16/17	Superior anterior most point of molar row
**Midline Landmarks**	**Anatomical Definition**
11	Mental spine and posterior midline bone-tooth junction
12	Anterior superior most point of incisor alveolar rim
19	Anterior most midline point of incisors

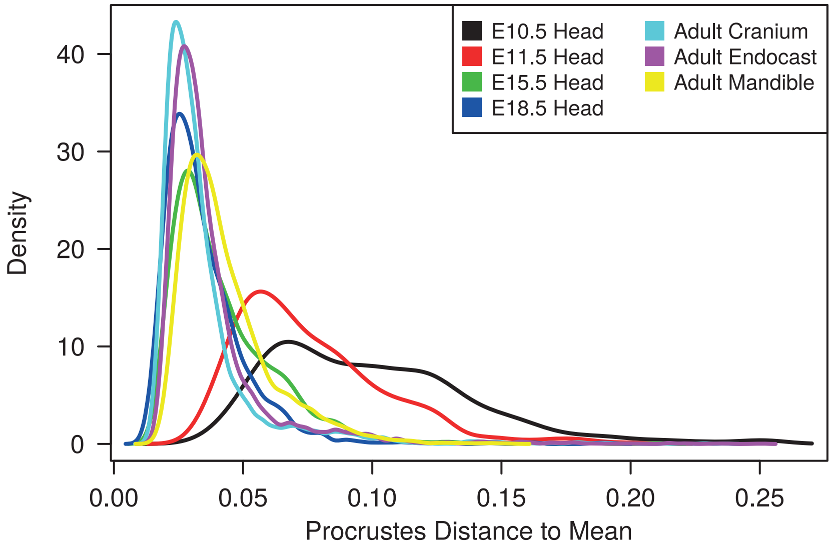

Figure S1. Density plot of Procrustes distances to the mean shape for each stage. These distributions were used to identify and eliminate global outliers.

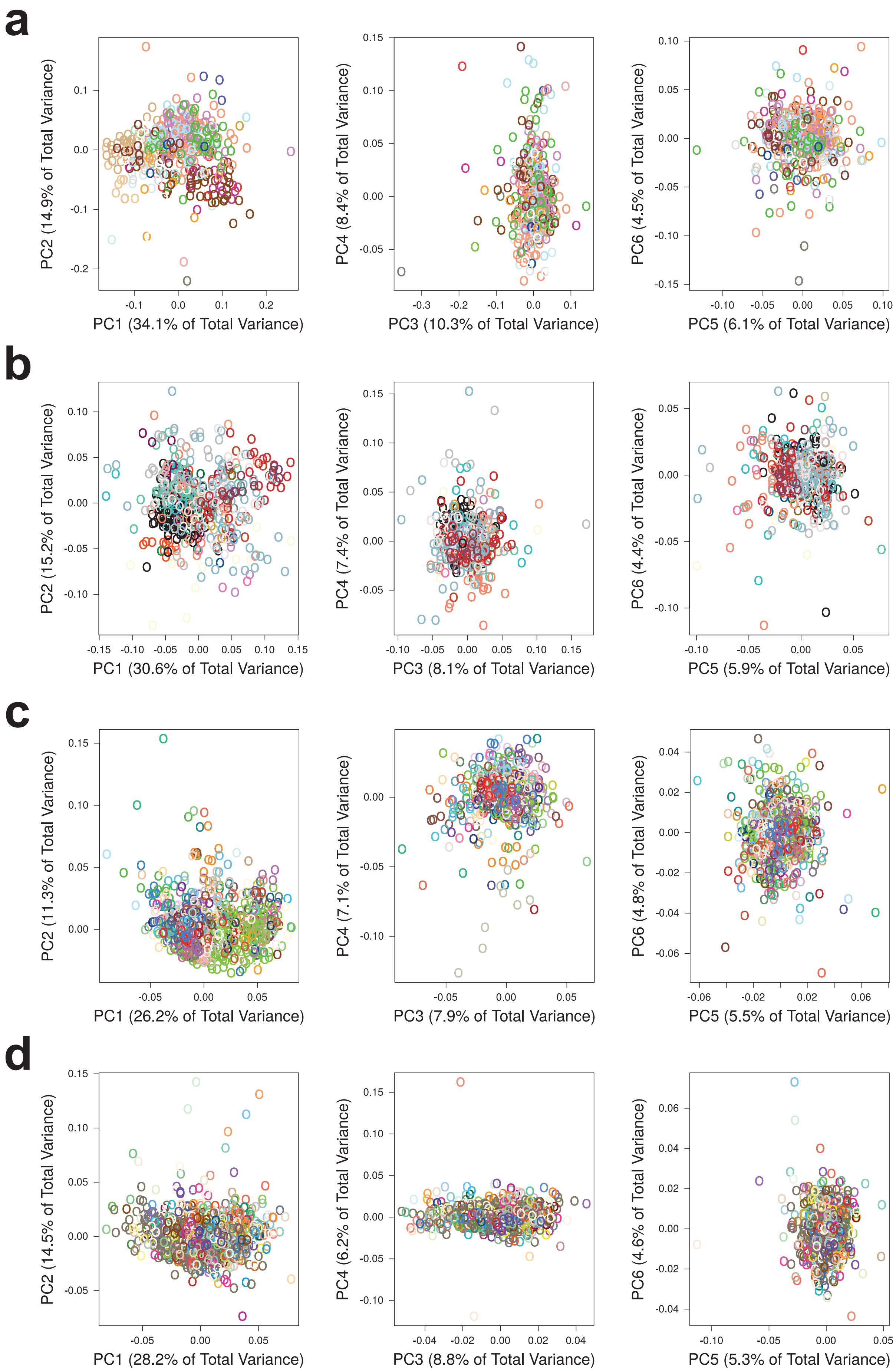

Figure S2. Principal Component Analysis of embryo landmark data. The first six PCs for (a) E10.5, (b) E11.5, (c) E15.5, and (d) E18.5 are shown. Each color represents a unique genotype within the stage. These data were used to identify and eliminate local outliers, as well as visualize stage-specific shape distributions.

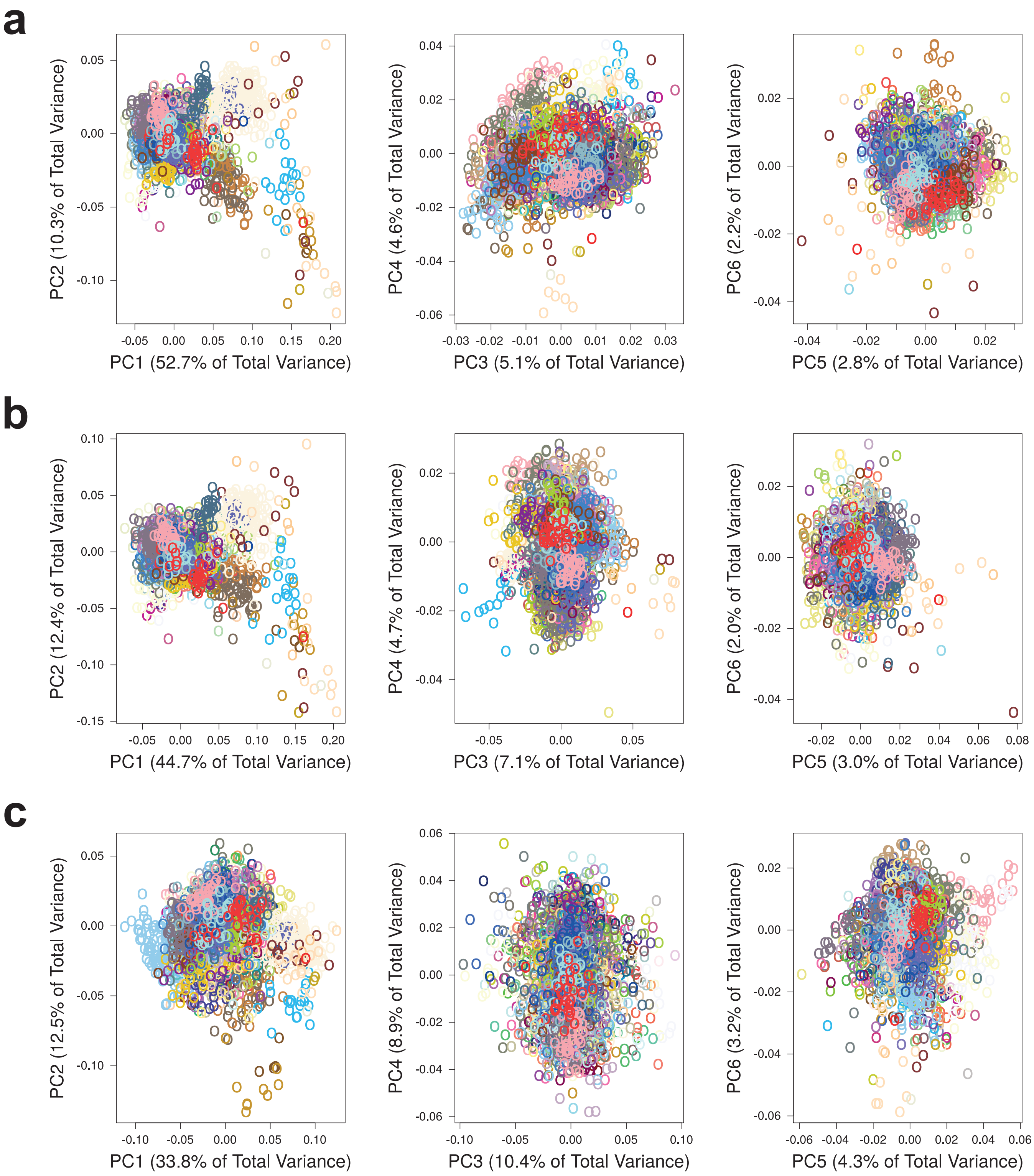

Figure S3. Principal Component Analysis of adult landmark data. The first six PCs for the (a) cranium, (b) endocast, and (c) mandible are shown. Each color represents a unique genotype. These data were used to identify and eliminate local outliers, as well as visualize the stage-specific shape distributions.

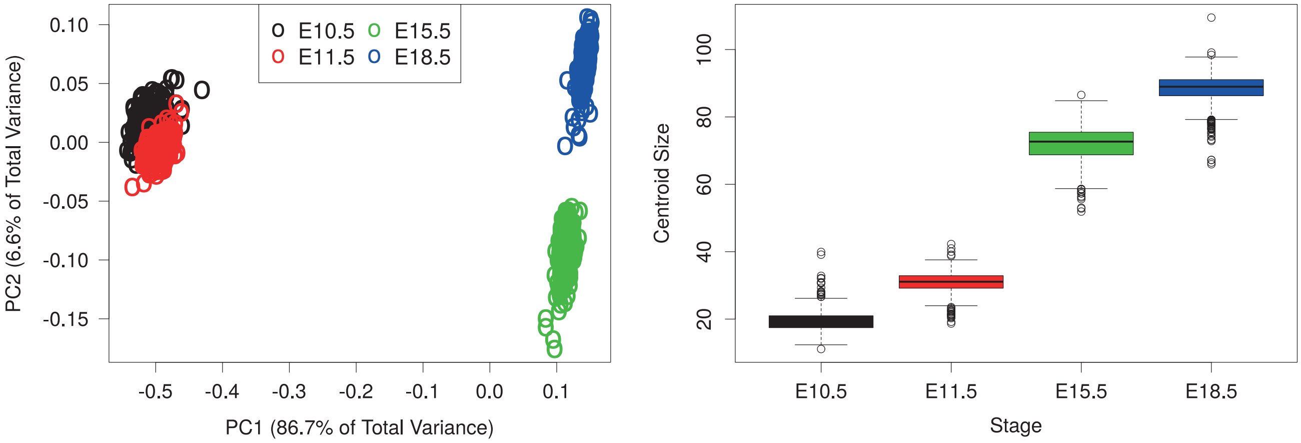

Figure S4. Generalized Procrustes Analysis of all embryo stages. For the sake of demonstration, an equivalent number of specimens were sampled from each stage (N=500) to not bias the mean. Left: Principal Component Analysis showing the large allometric effect along PC1. Right: Boxplots of centroid size obtained from the Procrustes superimposition.
